# Alternative host shapes transmission and life‐history trait correlations in a multi‐host plant pathogen

**DOI:** 10.1111/eva.13672

**Published:** 2024-03-10

**Authors:** Hanna Susi

**Affiliations:** ^1^ Research Centre for Ecological Change, Organismal and Evolutionary Biology Research Programme, Faculty of Biological and Environmental Sciences University of Helsinki Helsinki Finland

**Keywords:** disease evolution, ecology, epidemiology, generalist pathogen, host–pathogen interaction, landscape

## Abstract

Most pathogens are generalists capable of infecting multiple host species or strains. Trade‐offs in performance among different hosts are expected to limit the evolution of generalism. Despite the commonness of generalism, the variation in infectivity, transmission, and trade‐offs in performance among host species have rarely been studied in the wild. To understand the ecological and evolutionary drivers of multi‐host pathogen infectivity and transmission potential, I studied disease severity, transmission dynamics, and infectivity variation of downy mildew pathogen *Peronospora sparsa* on its three host plants *Rubus arcticus*, *R. chamaemorus*, and *R. saxatilis*. In a survey of 20 wild and cultivated sites of the three host species, disease severity varied by host species and by host population size but not among wild and cultivated sites. To understand how alternative host presence and plant diversity affect transmission of the pathogen, I conducted a transmission experiment. In this experiment, alternative host abundance and plant diversity together modified *P. sparsa* transmission to trap plants. To understand how resistance to *P. sparsa* varies among host species and genotypes, I conducted an inoculation experiment using 10 *P. sparsa* strains from different locations and 20 genotypes of the three host species. Significant variation in infectivity was found among host genotypes but not among host species. When trade‐offs for infectivity were tested, high infectivity in one host species correlated with high infectivity in another host species. However, when pathogen transmission‐related life‐history correlations were tested, a positive correlation was found in *R. arcticus* but not in *R. saxatilis*. The results suggest that host resistance may shape pathogen life‐history evolution with epidemiological consequences in a multi‐host pathogen.

## 1 INTRODUCTION

Many pathogens are generalists with the ability to infect multiple host species (Woolhouse et al., 2001). Generalism is common in all pathogen taxa, as exemplified by the malaria parasite *Plasmodium falciparum* (Hellgren et al., 2009), the SARS‐CoV‐2 virus (Damas et al., 2020), the white mold fungus *Sclerotinia sclerotiorum* (Bolton et al., 2006), and the bacterium *Pseudomonas syringae* (Barrett et al., 2009). The ability to infect multiple host species promotes the widespread dispersal of pathogens and their survival in one host while another goes locally extinct (Leggett et al., 2013). Disease symptoms may also differ among host species (Saikkonen et al., 2016). These characteristics make the management of generalist pathogens challenging and, consequently, their emergence can represent a widespread threat to the health of humans, crops, and ecosystems (Maloney et al., 2005). Moreover, increasing species introductions into new areas, including novel crops, and increased encounters between wild and cultivated species, have augmented the likelihood of emergence of multi‐host pathogens (Jeger, 2022). Despite such risks, surprisingly little is known about the epidemiology and evolution of multi‐host pathogens in wild and domesticated hosts (Barrett et al., 2009; Rigaud et al., 2010).

Overcoming diverse host resistance mechanisms is expected to be costly and limit the evolution of a generalist strategy (Leggett et al., 2013; Ravigné et al., 2009). The strength of inherent trade‐offs may define whether selection favors host specialists or generalists (Ravigné et al., 2009). Weak trade‐offs are expected to limit the evolution of specialists and strong trade‐offs are the evolution of generalists. There may, for example, be a trade‐off between specialization, allowing optimal acquisition of resources from just one to a few hosts, and more generalized but suboptimal resource acquisition from many hosts (Ravigné et al., 2009). In generalist pathogens, there may also be trade‐offs in performance among hosts, such that high performance in one host results in decreased performance in others (Joshi & Thompson, 1995; Kawecki, 1994), although the evidence for such costs is mixed (Leggett et al., 2013). Generalism might also accrue costs if high infectivity among host species limits pathogen performance within host individuals (Agudelo‐Romero & Elena, 2008). The underlying hypothesis is that if a pathogen can infect many hosts, it does not need to reach a high density within host individuals to persist (Combes, 1997). However, Hellgren et al. (2009) showed that pathogens with a high among‐species infectivity were also capable of reaching high densities within host individuals. Similarly, in a study of the multi‐host fungal pathogen *Microbotryum*, Bruns et al. (2021) found that pathogens with high intraspecific infectivity were capable of high sporulation in their shared host species. In addition, infecting novel host species allows pathogens to escape host resistance mechanisms (Barrett et al., 2009; Thines, 2019). It has even been suggested that life‐history trait correlations may be altered in novel hosts, that is, hosts in which the pathogen did not evolve (Thines, 2019), leading to a situation where pathogens may persist within hosts with low transmission and causing minimal harm (Saikkonen et al., 2016). In some cases, virulence (measurable harm to the host (Surico, 2013)) and transmission may be completely decoupled, for example, in the case of runaway virulence, where low transmission and disproportionately high harm co‐occur (Nelson & May, 2017; Rigaud et al., 2010; Woolhouse et al., 2001).

Additional important drivers of the evolution and epidemiology of multi‐host pathogens are community context and environmental variability (Gilligan & van den Bosch, 2008; Haas et al., 2011; Woolhouse et al., 2001). Alternative hosts may shape the evolution of virulence and the epidemiology of pathogens. The presence of highly susceptible reservoir host species may result in higher disease loads and also in more resistant hosts as shown in experiments on the *Barley yellow dwarf virus* and *Avena fatua* (Power & Mitchell, 2004). In an environment containing abundant alternative hosts that vary in their susceptibilities to the pathogen, increased infectivity is expected, whereas lower infectivity is expected when a single susceptible host species is present (Frank, 1992). In addition to the availability of susceptible hosts, overall community diversity can shape the transmission of multi‐host pathogens (Haas et al., 2011). In specialist pathogens, high diversity in the community in which the host is embedded is expected to have a diluting effect on disease transmission (Keesing et al., 2006, 2010; Rohr et al., 2020). Such a dilution effect has often been observed in plant communities (Civitello et al., 2015), but environmental variation may change this effect (Liu et al., 2020). An alternative scenario is amplification, especially in the case of multi‐host parasites, in which an increase in the number of competent hosts accelerates parasite spread (Begon, 2008; Johnson et al., 2016). However, asymmetry in host compatibility adds yet another layer of complexity to disease transmission within the community context (Haas et al., 2011; Pedersen & Fenton, 2007). Variation in resistance within and among species may thus have profound effects on the epidemiology and emergence of multi‐host pathogens (Woolhouse et al., 2001).

When novel crop species are taken into cultivation or a crop is cultivated in a new area, generalist pathogens existing in the same or alternative hosts in the wild may infect the cultivated plants leading to severe crop losses (Jeger, 2022). Understanding the host ranges, epidemiology, and transmission dynamics of local pathogen species is essential to prevent the emergence of pathogens in a changing crop selection (Jeger, 2022). *Rubus arcticus*, the arctic bramble, a berry crop with high‐value yields, has been cultivated in Finland since the 1970s. However, a downy mildew pathogen disease (“dryberry” disease) caused by the oomycete *Peronospora sparsa* has caused devastating losses of harvest (Koponen et al., 2000). Outbreaks of downy mildew cause losses of up to 50% in marketable yield due to drying of the developing berries (Koponen et al., 2000). The pathogen favors a cool and moist climate and spreads via air and water droplets. Current control methods rely on fungicides as there are no resistant cultivars available (Kostamo et al., 2015; Parikka et al., 2016). *P. sparsa* is a multi‐host pathogen (Hukkanen et al., 2006); in addition to wild *R. arcticus*, its other potential host species, the cloudberry, *Rubus chamaemorus*, and stone bramble, *Rubus saxatilis*, grow commonly in forests and roadsides adjacent to plantations, making pathogen transmission between fields and wild plants a challenge for disease management. The pathogen has been previously reported in *R. chamaemorus* and *R. arcticus* but not in *R. saxatilis* (Koponen et al., 2000). Laboratory inoculations have confirmed that *P. sparsa* infects *R. arcticus* and *R. chamaemorus* but no inoculation trials on *R. saxatilis* have been reported (Koponen et al., 2000). Currently, the pathogen's ecology and adaptation to its host species are poorly understood.

Here, I studied the host range, transmission dynamics, and life‐history correlations of the oomycete multi‐host pathogen *P. sparsa* on its three *Rosaceae* host species *R. arcticus*, *R. chamaemorus*, and *R. saxatilis* by conducting a field survey, a transmission experiment, and a laboratory inoculation experiment. To understand the pathogen's natural host range and prevalence in these three host species, I used PCR tests to detect the presence of the pathogen, and I recorded symptoms of infection in plant samples collected from *Rubus* populations at 20 sites. Transmission was experimentally investigated by placing cloned, pathogen‐free *R. arcticus* plants into 20 *R. arcticus* populations with naturally varying frequencies of *R. saxatilis*. To understand *P. sparsa* performance in the three host species, a laboratory inoculation trial using 10 *P. sparsa* strains originating from wild sites was set up. Specifically, the aims of the study were to test: (1) Does *P. sparsa* infect *R. saxatilis* and *R. chamaemorus* in the wild? (2) How common are symptoms on the three host species in wild versus cultivated populations? (3) Does transmission of *P. sparsa* vary with plant diversity and host abundance? I expect to find a dilution effect of plant diversity on transmission (Liu et al., 2020) and that transmission increases as a function of host abundance (Frank, 1992; Woolhouse et al., 2001). (4) What determines the outcome of *P. sparsa* inoculation: Pathogen genotype, host species, or host genotype? I expect *P. sparsa* performance to vary by host species in the laboratory. (5) Are there trade‐offs between pathogen life‐history traits? I expect that high performance in one host comes at a cost of poorer performance in another (Kawecki & Ebert, 2004).

## 2 MATERIALS AND METHODS

### 2.1 The hosts and the pathogen

*R. arcticus* L is a perennial diploid plant with a native distribution spanning subarctic Eurasia, Asia, and North America. The plant is an obligate out‐crosser and spreads vegetatively via rhizomes (Tammisola, 1988). *R. arcticus* is cultivated for commercial use in Finland but its yields have been severely damaged by downy mildew disease caused by *P. sparsa* (Koponen et al., 2000). *R. chamaemorus* is a perennial dioecious wild plant native to northern hemisphere. It occurs in mountainous areas and moorlands. Despite some attempts to cultivate the plant for its berries and leaves, *R. chamaemorus* is not widely cultivated. *R. saxatilis* is a perennial plant distributed in temperate regions in Eurasia commonly occurring in forests and field sides. The plant is not commercially cultivated. *R. arcticus* and *R. saxatilis* are phylogenetically close relatives belonging to *Cylactis* subgenus, whereas *R. chamaemorus* belongs to *Chamaemorus* subgenus (Sobczyk, 2018).

*P. sparsa* is an obligate biotroph (Thines & Choi, 2016) and spreads via several asexual reproductive cycles during the growing season (Kostamo et al., 2015). Asexual spores are spread by air and water and germination occurs in moist and cool conditions (Aegerter et al., 2003). *P. sparsa* is divided into two subspecies, one of which infects the genus *Rosa* and another the genus *Rubus* (Thines & Choi, 2016).

### 2.2 Survey and detection of *P. sparsa* in cultivations and wild sites

To investigate the prevalence and host range of *P. sparsa* in naturally occurring *Rubus* species in Finland, I surveyed 20 sites containing either one or two *Rubus* species (15 natural sites and five plantations; Table S1; Figure S1a) across Finland in late August 2019. At each site, *P. sparsa* infection severity in 10–30 plants of each *Rubus* species was estimated as symptomatic area as a proportion of total leaf area per plant. Host plant population size at each site was estimated as the area covered by the host species in square meters. Infection prevalence was estimated as the percentage of infected hosts with visible symptoms. To confirm that *P. sparsa* was the causal agent of disease symptoms, a 2 cm^2^ piece of a symptomatic leaf was collected into a microtube and stored at −80°C until DNA extraction. Samples were collected from 10 plants of each species at each site.

In the laboratory, DNA was extracted from the samples using the CTAB method (Lodhi et al., 1994). The yield and quality of DNA were measured using Nanodrop. The PCR reaction was set up using primers P1 and P2 (Hukkanen et al., 2006) as follows: 10 μL PCR mix included 0.5 volume GoTaq® Green Master Mix (Promega), 1 μL primer P1 (10 μM), 1 μL primer P2 (10 μM), 3 μL MilliQ water, and 1 μL template. The reaction cycle was 92°C for 2 min, 30 cycles at 92°C for 30 s, 56°C for 30 s, and 72°C for 30 s with final extension at 72°C for 5 min. The PCR product was subjected to gel electrophoresis and visualized using BioRad Gel Doc X System.

### 2.3 Transmission experiment

Transmission dynamics were observed by placing *R. arcticus* trap plants in 20 *R. arcticus* sites with varying coverages of *R. saxatilis* and *R. arcticus*. Plant material for the transmission experiment was obtained by cloning small *R. arcticus* plants from rhizomes in an insect‐free greenhouse in 16:8 day:night conditions at 17 ± 2°C for 4 months. The clones were separated from the plant as terminal buds of the rhizome and placed in separate 8 × 8 cm pots filled with a mixture of 25% potting soil, 25% lightweight expanded clay aggregate, and 50% peat. After cloning, the dormant buds were kept at +4°C for 4 months until the experiment. Thus, all plants were in the same early stages of development when transplanted into the field sites. Three pathogen‐free *R. arcticus* genotypes were used in the experiment, commercial cultivar *Pima*, and wild genotypes G12 and G13 originating from Muuruvesi and Viinikka, respectively. To understand how the presence of alternative hosts and surrounding plant diversity affect *P. sparsa* transmission and within‐host infection severity, I set up a field experiment using 600 plants distributed among 20 sites (30 plants per site). At 10 of these sites, only *R. arcticus* was present, and in the remaining sites, *R. arcticus* co‐occurred with either *R. chamaemorus* (1 site) or *R. saxatilis* (9 sites). The FinBIF Database (https://laji.fi/taxon/MX.mesimarja, accessed 14.06.2021) was used in site selection and in determining the presence of *Rubus* species. The experiment was set up in mid‐June 2021. At each site, 30 trap plants in pots, each representing a single *R. arcticus* genotype (*Pima*, G12 or G13), were placed randomly within a 0.5 m distance of growing *Rubus* plants. The trap plants were left to grow at the sites for 7 weeks, after which they were revisited, their leaves were counted, and leaf infection status (0 = no infection; 1 = infection) was observed and later confirmed by microscopy, if ambiguous. When an infection was observed in a trap plant, the number of infected leaves was counted. To evaluate the association between *P. sparsa* transmission and plant diversity, all vascular plant species growing within 3–6 1 m^2^ vegetation plots were identified at each site (the precise number of plots surveyed was defined by the total area of the site). The coverage as percentage of the plot area of each vascular plant species was estimated in each plot and the coverage of each species at the site level was calculated as the average of its coverage within the vegetation plots in the site. *Shannon*'s index of vascular plant diversity (Shannon & Weaver, 1949) at each site was calculated as:

$$H=-\mathrm{SUM}\left[\left(\mathrm{pi}\right)*\ln\left(\mathrm{pi}\right)\right]$$

where pi is the coverage of a plant species as the summed occurrence (0 = species absent in the plot; 100 = species covers the whole plot area) of species/total number of vegetation plots within each site.

### 2.4 Inoculation experiment

To confirm the host range of *P. sparsa* and to compare its performance and life‐history trait correlations among *R. arcticus*, *R. saxatilis*, and *R. chamaemorus*, an inoculation experiment was set up. Ten *P. sparsa* strains (Table S2) originating from different sites were used in the experiment. Infected *R. arcticus* leaves were collected from the sites, placed on Petri dishes, and brought to the laboratory. Spores of the pathogen were then inoculated onto young, detached leaves of susceptible *R. arcticus* plants on Petri dishes. The inoculated leaves were maintained in 16:8 day:night conditions at 17 ± 2°C for 2 weeks after which they were transferred onto new leaves. The plant material for the inoculation experiment consisted of 20 host genotypes (11 *R. arcticus* genotypes, 4 *R. chamaemorus* genotypes, and 5 *R. saxatilis* genotypes; Table S3) that were inoculated with the 10 *P. sparsa* strains resulting in 200 host genotype–pathogen genotype combinations. Each combination was replicated three times leading to 600 inoculations in total. Detached leaves placed on moist filter paper on Petri dishes were inoculated with conidial spores from 1 cm^2^ lesions by evenly brushing the spores (approx. 100 spores) on a leaf with a moist paintbrush. Pathogen development was observed daily under a dissecting microscope from the 7th‐day post‐inoculation (DPI) until day 21 DPI. Young *Rubus* leaves generally keep well on moist Petri dishes at 17°C with artificial lightning for up to 28 days. Infectivity was recorded as 0 = no infection and 1 = infection (lesion development observed). I measured two components of pathogen transmission potential: time to pathogen sporulation and pathogen lesion development on day 21 DPI. The first day on which spores were observed was considered the day of sporulation. Pathogen sporulation was measured on a scale from 0 to 4: 0 = no mycelium and 1 = only mycelium; 2 = mycelium and sparse sporulation visible under the microscope only; 3 = abundant sporulation and colony size <0.5 cm^2^; and 4 = abundant sporulation and colony size >0.5 cm^2^ (Bevan et al., 1993).

### 2.5 Statistical analyses

#### 2.5.1 Analyses of the survey results in cultivated and wild sites

To understand the sources of variation in disease prevalence and severity across the 20 cultivated and wild *Rubus* sites surveyed in 2019 (Figure S1), I ran a set of generalized linear mixed models (GLMM) in SAS Proc Glimmix (SAS Institute). First, I ran a model to test whether *R. arcticus* and *R. chamaemorus* growing in cultivated and wild sites differ in their disease prevalence measured as the proportion of diseased plants in each site (model a in Table 1). *R. saxatilis* was excluded from this model because it is never cultivated. Disease prevalence was coded as a binomial response variable. Plant population size was included as a continuous explanatory variable, and focal host species and cultivated versus wild as class explanatory variables. Site ID was treated as a random variable. Secondly, I fit another model with the same structure to understand the drivers of disease severity with a beta error distribution (model b in Table 1). In these two models, I test for an interaction between focal host species and cultivated versus wild. The final models were selected using the Akaike information criteria (AIC) (Symonds & Moussalli, 2011).

**TABLE 1:** The details of the generalized linear (mixed) models A–J used in the study.

Model	Response variable	Variable type	Source of variation	Error distribution	Interactions used in model selection	AIC
a	Survey in cultivated and wild sites, cultivated vs. wild					
	Disease prevalence	Class	**Focal species**	**Binomial**	**None**	**37.04**
			Cultivated vs. wild		Focal species × Cultivated vs. wild	42.68
		Continuous	Cov.^Focal species^			
		Random	Site ID			
b	Survey in cultivated and wild sites, cultivated vs. wild					
	Disease severity	Class	Focal species	**Beta**	**None**	**−1042.1**
			Cultivated vs. wild		Focal species × Cultivated vs. wild	−1040.46
		Continuous	Cov.^Focal species^			
		Random	Site ID			
c	Survey in cultivated and wild sites, alternative host presence					
	Disease prevalence	Class	Focal species	**Binomial**	**None**	**45.43**
			Alternative host species		Focal host species × Alternative host species	47.43
		Continuous	Cov. ^Focal species^			
		Random	Site ID			
d	Survey in cultivated and wild sites, alternative host presence					
	Disease severity	Class	Focal species	**Beta**	**None**	**−1480.03**
			Alternative host species		Alternative host species × Cov.^Focal species^	−1478.63
		Continuous	Cov.^Focal species^			
		Random	Site ID			
e	Transmission experiment					
	Disease prevalence	Class	Genotype	**Binomial**	None	369.15
		Continuous	Cov.^R. sax^		Cov.^R. sax^ × Plant diversity	360.44
			Cov.^R. arct^		Cov.^R. arct^ × Plant diversity	363.2
			Plant diversity		Cov.^R. arct^ × Plant diversity, Cov.^R. arct^ × Cov.^R. sax^	360.44
			Leaf number		Cov.^R. arct^ × Cov.^R. sax^	362.75
		Random	Population		**Cov.** ^**R. arct**^ **× Plant diversity, Cov.** ^**R. sax**^ **× Plant diversity**	**354.35**
f	Transmission experiment					
	Disease severity	Class	Genotype	**Binomial**	None	373.16
		Continuous	Coverage^R. sax^		Cov.^R. sax^ × Plant diversity	369.14
			Coverage^R. arct^		Cov.^R. arct^ × Plant diversity	373.3
			Plant diversity		Cov.^R. sax^ × Plant diversity, Cov.^R. arct^ × Cov.^R. sax^	371.11
			Leaf number		Cov.^R. arct^ × Plant diversity, Cov.^R. arct^ × Cov.^R. sax^	373.75
		Random	Population		Cov.^R. arct^ × Cov.^R. sax^	372.9
					**Cov.** ^**R. arct**^ **× Plant diversity, Cov.** ^**R. sax**^ **× Plant diversity**	**367.22**
g	Inoculation experiment					
	Infectivity	Class	Species	**Binomial**	**None**	**181.32**
			Genotype (Species)			
			*P. sparsa strain*			
h	Inoculation experiment					
	Sporulation abundance	Class	Species	**Gaussian**	**None**	**124.6**
			Genotype (Species)			
			*P. sparsa strain*			
i	Laboratory experiment					
	Speed to sporulation	Class	Species	**Gaussian**	**None**	**44.03**
			Genotype (Species)			
			*P. sparsa strain*			
j	Laboratory experiment					
	Sporulation abundance	Class	Species	**Gaussian**	None	98.88
			Genotype (Species)		Speed to sporulation × (Genotype) Species	103.49
		Continuous	Speed to sporulation		**Speed to sporulation × Species**	**92.59**
		Random	*P. sparsa strain*			

*Note*: When multiple models were tested, Akaike information criteria (AIC; smaller is better) values are reported and the best models are indicated in bold.

Thirdly, I fit a model using the full data set collected from the wild and cultivated sites of the three *Rubus* species to test why there is variation in disease prevalence (model c in Table 1). Disease prevalence per *Rubus* host species at each site was coded as a binomial response variable. Host population size, measured as the coverage of the focal host species in square meters, was used as a continuous explanatory variable. Focal host species and the presence of alternative host species (1 = alternative host present, 0 = no alternative host present) were used as class explanatory variables. Site ID was treated as random variable. A binomial error distribution was assumed. Finally, I fit a model with identical structure to understand the effects of host species, population size, and alternative host presence on disease severity measured as the proportion of symptomatic leaf area in each surveyed plant (model d in Table 1). In this model, a beta error distribution was assumed. In both models, I tested for an interaction between the focal host species and the presence of alternative host species. The final models were selected using the AIC of the model.

#### 2.5.2 Transmission experiment analyses

To explore the drivers of transmission of *P. sparsa* in sites with varying prevalences of *Rubus* species and plant diversity, two GLMMs were set up in SAS Proc Glimmix (SAS Institute). The data collected in the transmission experiment in 2021 was used in these analyses. In the first model (model e in Table 1), I used the infection status of each trap plant (1 = infected, 0 = no infection) as a binomial response variable. Plant diversity (*Shannon* diversity) of plants, the coverages of *R. arcticus* and *R. saxatilis*, and the plant size (as number of leaves) were used as continuous explanatory variables. Trap plant genotypes were used as class explanatory variables. A binomial distribution of error was assumed. The site ID was used as a random variable. To understand the drivers of within‐host spread of the pathogen, I fit another model (model f in Table 1) with similar structure but used the proportion of infected leaves within trap plant as binomial response variable. In this model, binomial distribution of error was assumed. In these models, the interactions among diversity of plants, *R. arcticus* coverage, and *R. saxatilis* coverage were tested. The final models were selected using the AIC of the model. The interaction results were visualized by dividing *R. arcticus* and *R. saxatilis* populations into small and large by their coverage. Both plant species occur in clonal growth form, particularly in small populations consisting of only few individuals (Eriksson & Bremer, 1993; Tammisola, 1988) and, thus a threshold of 2.0 m^2^ was chosen to split small and large populations to reflect potential genetic diversity of the host populations.

The relationships among the site variables in the transmission experiment (plant diversity, *R. arcticus* coverage, and *R. saxatilis* coverage) and latitude were furthermore summarized via a principal component analysis (PCA) using the *vegan* R package (Oksanen et al., 2018). Before running the ordination, the matrix of variables entered into the PCA was scaled using *scale* function.

#### 2.5.3 Inoculation experiment analyses

To assess the effects of *P. sparsa* strain, host species and genotype on infectivity, speed to sporulation, and sporulation abundance three models were set up using generalized linear model framework in SAS Proc Glimmix (SAS Institute). First, variation in infectivity was modeled using the infection outcome (0 = no infection; 1 = infection) as a binomial response variable (model g in Table 1). *P. sparsa* strain, host species, and host genotype nested under host species were used as class explanatory variables. The second model (model h in Table 1) with a similar structure was set up to assess the speed of sporulation. Time to sporulation was measured as the day of sporulation subtracted from 21 days (the end of the experiment), was log‐transformed, and was modeled as a continuous response variable. A Gaussian error distribution was assumed. The third model (model i in Table 1) with a similar structure was fitted to predict variation in *P. sparsa* sporulation on a scale from 0 to 4. In this model, a Gaussian error distribution was assumed.

#### 2.5.4 Life‐history trait correlation analyses

Lastly, I set up four models to test whether there are potential trade‐offs between pathogen life‐history traits and whether host species affect potential life‐history trait correlations. First, to test whether high infectivity in one host species has a cost as reduced infectivity in other host species, three models in Proc REG in SAS (SAS Institute) using the mean infectivity of each *P. sparsa* strain on each host species as the response variable were run. Secondly, to understand whether fast sporulation is related to a high sporulation abundance at the end of the experiment and whether host species influences this relationship, I ran a GLMM in Proc Glimmix in SAS (SAS Institute) using the mean sporulation stage of the pathogen on each host genotype as a continuous response variable (model j in Table 1). Speed to sporulation was used as a continuous explanatory variable. Host species and host genotype nested under host species were used as class explanatory variables. *P. sparsa* strain was included as a random variable. A Gaussian error distribution was assumed. Interactions among host species, host genotype nested under host species, and speed to sporulation were tested, and the final model was selected that minimized the AIC.

## 3 RESULTS

### 3.1 Disease prevalence and severity in surveys of *Rubus* at wild and cultivated sites

**FIGURE 1:**
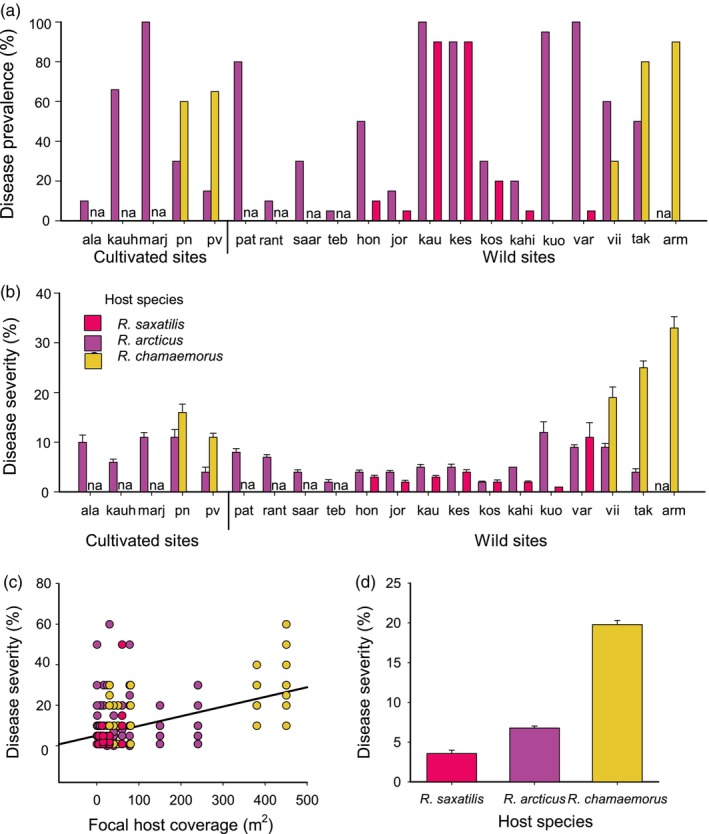
Variation in *Peronospora sparsa* symptoms in three *Rubus* hosts in the wild and cultivated sites. The variation in *P. sparsa* (a) disease prevalence (percentage of hosts infected in a population) and (b) severity (as percentage of infected leaf area) on *Rubus arcticus*, *R. chamaemorus*, and *R. saxatilis* in 20 sites in 2019. Letters na indicate that there were no alternative host species present in the population. (c) The relationship between disease severity and host population size. (d) The variation in disease severity among the three host species.

**FIGURE 2:**
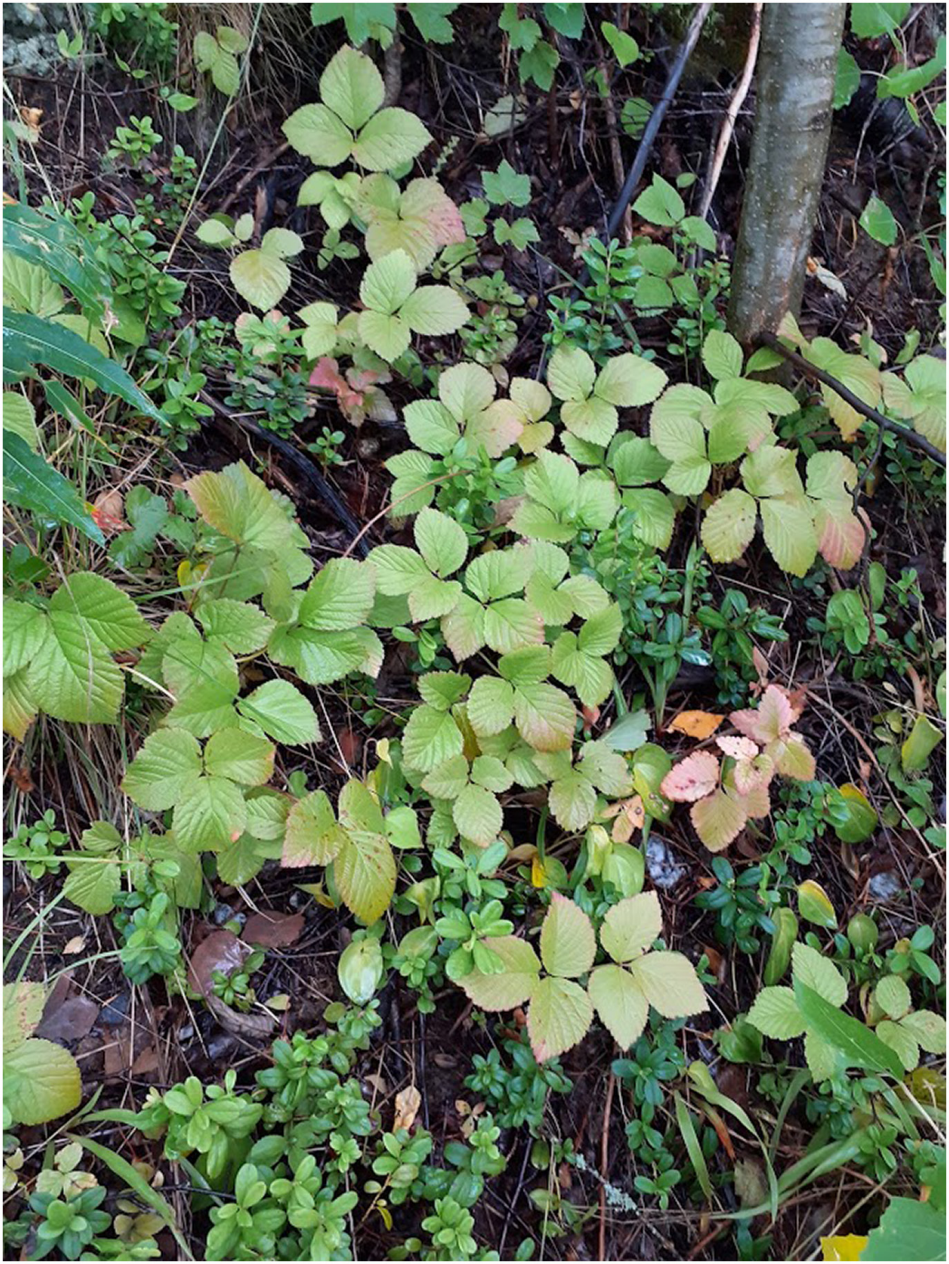
*Rubus saxatilis* plants with mild symptoms of *Peronospora sparsa* growing in a natural population.

*P. sparsa* was detected at the surveyed field sites not only in both wild and cultivated *R. arcticus* but also in a novel host plant in the wild, *R. saxatilis* (Figures 1a,b and 2). In the infected sites, a high disease prevalence was found in *R. arcticus* (58%), *R. chamaemorus* (64%), and *R. saxatilis* (36%). However, host species identity, the presence of alternative host species, or population size explained the observed among‐site variation in disease prevalence (Table 2). The host species differed in their disease severities. *R. chamaemorus* had the highest disease severity (19.6% leaf area symptomatic), while the two other hosts had milder symptoms *R. arcticus* (6.8%) and *R. saxatilis* (3.6%; Table 2; Figure 1d and Figure S1b–d). Population size was positively correlated with disease severity (Table 2; Figure 1c) but alternative host presence did not alter disease severity (Table 2).

**TABLE 2:** Factors affecting *Peronospora sparsa* disease severity and prevalence in 20 sites surveyed for *Rubus arcticus*, *R. chamaemorus*, and R. *saxatilis* in Finland in 2019.

Effect	Cultivated vs. wild host								Alternative host							
	a—Disease prevalence				b—Disease severity				c—Disease prevalence				d—Disease severity			
	Num DF	Den DF	*F*	*p*	Num DF	Den DF	*F*	*p*	Num DF	Den DF	*F*	*p*	Num DF	Den DF	*F*	*p*
Focal host coverage	1	**9**	1.08	0.37	**1**	**315**	14.7	0	1	9	0.39	0.55	1	425	16.1	<0.0001
Cultivated vs. wild	1	9	0.01	0.91	**1**	**315**	1.14	0.29		**–**	**–**	**–**		**–**	**–**	**–**
Focal host species	**2**	**9**	0.15	0.87	**2**	**315**	**14.7**	**<0.0001**	**1**	9	0.32	0.58	1	**425**	**17.4**	**<0.0001**
Alternative host species		**–**	**–**	**–**		**–**	**–**	**–**	1	9	0.85	0.38	**1**	425	2.71	0.1

*Note*: The results were analyzed with generalized linear mixed models. Statistically significant (*p* < 0.05) results are shown in bold. The letters a–d refer to the models described in Table 1.

When an analysis was run on a subset of sites with the two cultivated hosts, *R. arcticus* and *R. chamaemorus*, no difference in disease prevalence was found between cultivated and wild sites, host species, or population sizes (Table 2). Cultivated and wild sites did not differ in their disease severity, but severity again differed among the host species and there was a positive correlation between disease severity and population size (Table 2).

### 3.2 Transmission experiment results

**FIGURE 3:**
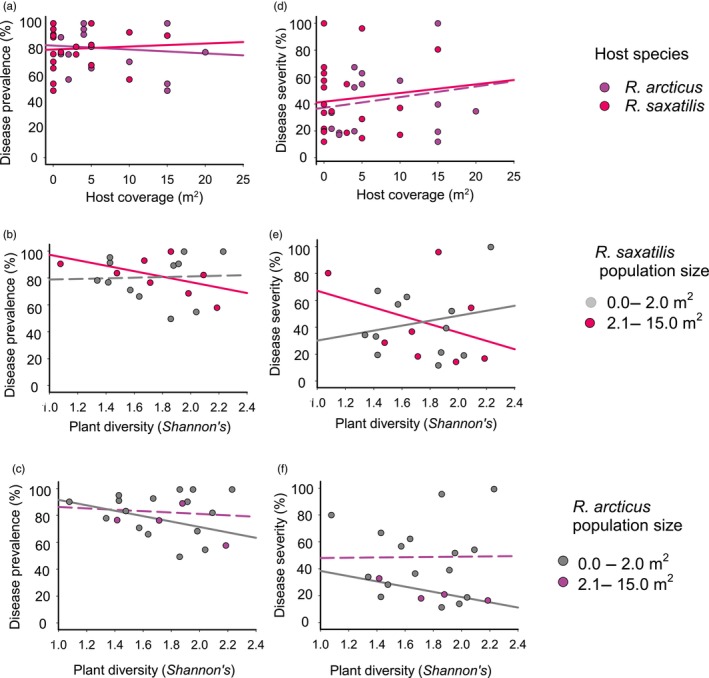
Variation in *Peronospora sparsa* transmission to *Rubus arcticus* trap plants (*n* = 493) in 20 sites with varying alternative host abundance. The relationship between *P. sparsa* disease prevalence and (a) *R. arcticus* and *R. saxatilis* population size, (b) plant diversity in small (<2.0 m^2^ coverage; grey circles) and large (>2.0 m^2^ coverage; red circles) *R. saxatilis* populations, (c) plant diversity in small (<2.0 m^2^ coverage; grey circles) and large *R. arcticus* (purple circles) populations. The relationship between *P. sparsa* disease severity (percentage of infected leaves within a trap plant) and (d) *R. arcticus* (purple circles) and *R. saxatilis* (red circles) coverage, and (e) plant diversity in small (<2.0 m^2^ coverage; grey circles) and large (>2.0 m^2^ coverage; red circles) *R. saxatilis* populations, and (f) plant diversity in small (<2.0 m^2^ coverage; grey circles) and large *R. arcticus* (>2.0 m^2^ coverage; purple circles) populations. Significant correlations are indicated with solid lines.

Altogether 78% (*n* = 466) of the *Rubus* trap plants survived throughout the 7‐week transmission experiment (20%–100% of plants survived within each site). Plant size, genotype, as well as *R. arcticus* and *R. saxatilis* coverage were all significantly associated with among‐site variation in disease prevalence in the trap plants (Table 3; Figure 3a). Plant size was positively related to infection prevalence. Infection prevalence was highest in plant genotype G12 and lowest in genotype G13. The host plant combination in the study sites was also linked to *P. sparsa* transmission. *R. saxatilis* population size (coverage) increased disease prevalence, whereas the opposite was true for *R. arcticus* (Table 3; Figure 3a). Plant diversity had no direct effect on disease prevalence (Table 3). However, there was a significant interaction between *R. saxatilis* coverage and plant diversity (Table 3; Figure 3b). There was a negative relationship between plant diversity and disease prevalence in sites with a higher *R. saxatilis* coverage (>2.0 m^2^), whereas in sites with little or no *R. saxatilis* (0–2.0 m^2^ coverage), no such relationship was found (Table 3; Figure 3b). There was contrasting interaction between *R. arcticus* population size and plant diversity (Table 3; Figure 3c). In sites with small *R. arcticus* populations (<2.0 m^2^ coverage), plant diversity was negatively correlated with disease prevalence but in large *R. arcticus* populations (>2.0 m^2^ coverage) no such relationship was seen (Table 3; Figure 3c).

**TABLE 3:** Factors affecting the transmission of *Peronospora sparsa* to *Rubus arcticus* trap plants in 20 sites in Finland.

Effect	e—Disease prevalence				f—Disease severity			
	Num DF	Den DF	*F*	*p*	Num DF	Den DF	*F*	*p*
Genotype	**2**	**429**	**6**	**0.0027**	2	343	2.52	0.0816
Coverage^*R. saxatilis*^	**1**	**429**	**8.78**	**0.0032**	**1**	**343**	**6.83**	**0.0093**
Coverage^*R. arcticus*^	**1**	**429**	**5.5**	**0.0194**	1	343	2.96	0.0864
Plan*t* diversity	1	**429**	0.13	0.7176	1	343	0.27	0.605
Leaf number	**1**	**429**	**26.32**	**<0.0001**	**1**	**343**	**8.07**	**0.0048**
Coverage^*R. saxatilis*^ × Plant diversity	**1**	**429**	**7.95**	**0.005**	**1**	**343**	**7.08**	**0.0082**
Coverage^*R. arcticus*^ × Plant diversity	**1**	**429**	**5.01**	**0.0257**	**1**	**343**	**4.15**	**0.0425**

*Note*: Plant size is the number of leaves of trap plants when the sites were revisited at the end of a 7‐week field experiment. The results were analyzed with generalized linear models. Statistically significant (*p* < 0.05) results are shown in bold. The letters e, f refer to the models described in Table 1.

When disease severity was analyzed, I found that *R. saxatilis* population size but not *R. arcticus* population size correlated positively with disease severity (Table 3; Figure 3d). The effect of plant diversity alone was not significant but was again mediated by *R. saxatilis* coverage (significant interaction of *R. saxatilis* coverage × plant diversity; Table 3; Figure 3e). In the sites with high *R. saxatilis* coverage, plant diversity correlated negatively with disease severity, whereas in the sites with no or low *R. saxatilis* coverage, the opposite was observed. There was also interaction between *R. arcticus* population size and plant diversity (significant interaction between *R. arcticus* coverage × plant diversity; Table 3; Figure 3f). In large *R. arcticus* populations, no correlation was observed but in small populations, a negative correlation between plant diversity and disease severity was observed. Large plants were less severely infected than small plants (Table 3), but the trap plant genotypes did not vary in their disease severity (Table 3). A PCA on the site variables (plant diversity, *R. arcticus* coverage, *R. saxatilis* coverage, and latitude) indicated that there were no strong correlations among them (Figure S2).

### 3.3 Inoculation experiment results

**FIGURE 4:**
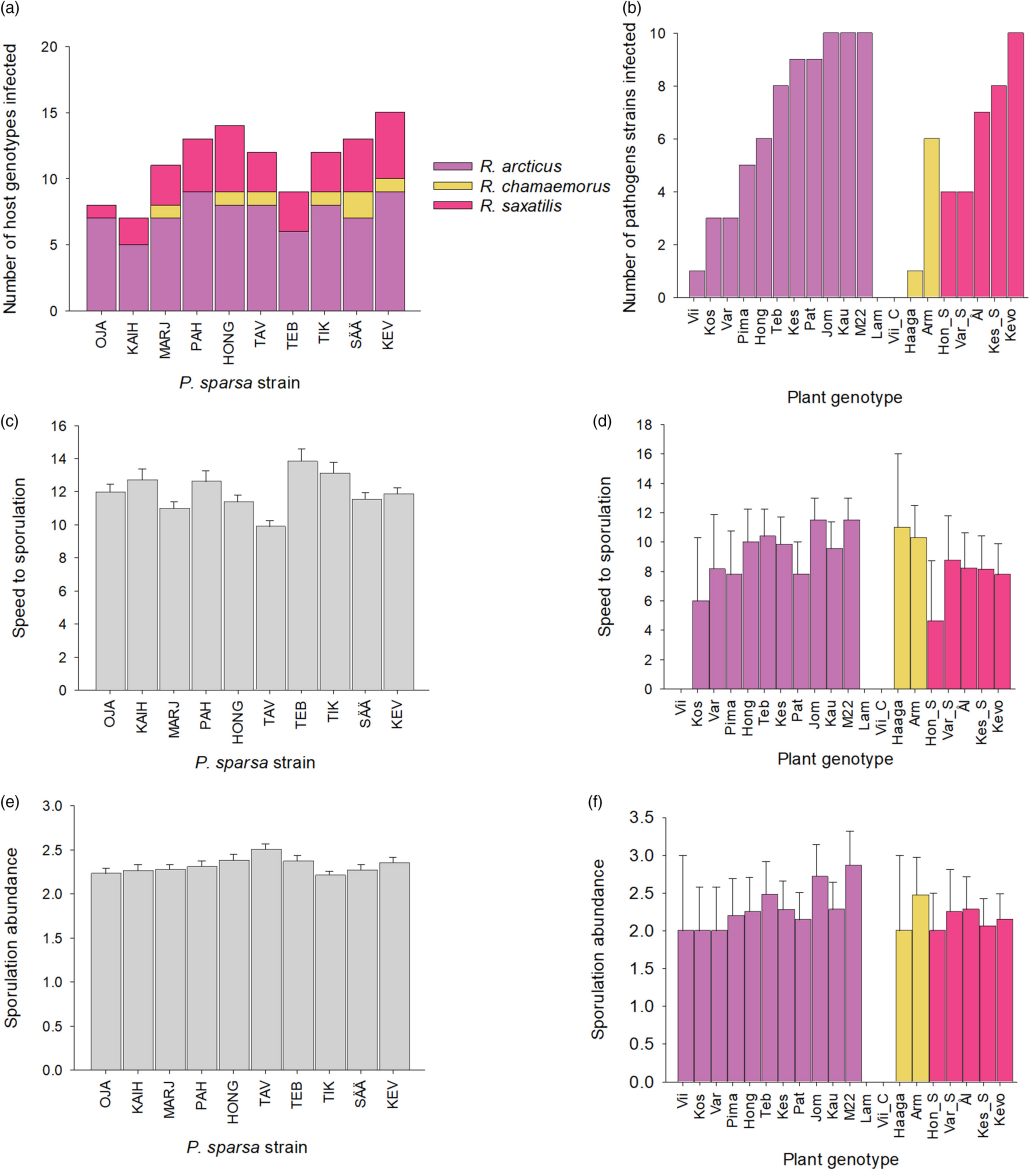
Variation in infectivity and transmission potential in an inoculation experiment on 10 *Peronospora sparsa* strains and 11 *Rubus arcticus*, 4 *R. chamaemorus*, and 5 *R. saxatilis* genotypes. (a) Variation among *P. sparsa* strains in their ability to infect host genotypes. (b) The variation among *Rubus* genotypes in the infectivity of *P. sparsa*. The variation in speed to first sporulation (21 days—the day when first sporulation was observed in microscopy) among (c) *P. sparsa* strains (across all genotypes) and (d) *Rubus* genotypes. The variation in sporulation abundance of (e) *P. sparsa* strains and (f) *Rubus* genotypes.

When pathogen infectivity (i.e., host susceptibility) was tested, there was significant variation among the *P. sparsa* strains (Table 4; Figure 4a). A generalist strategy was common among the studied pathogen strains as six of the 10 *P. sparsa* strains were able to infect all three host species (Figure 4a). Infectivity among the 20 plant genotypes varied from 35% to 75% (Figure 4a). Two of the *R. chamaemorus* genotypes were completely resistant to *P. sparsa* infection (Figure 4b). Host genotypes within host species differed significantly in their susceptibility to infection but host species did not explain a significant amount of variation (Table 4; Figure 4b). The susceptibility of the plant genotypes to the experimental pathogen strains ranged from 0% to 100% (Figure 4b). When the factors driving transmission potential, that is, speed to sporulation and sporulation abundance were analyzed, there were significant differences between host genotypes within species but neither pathogen strain nor host species had significant effects on these life‐history traits (Table 4; Figure 4c–f).

**TABLE 4:** Factors affecting the infection success, speed to sporulation, and sporulation abundance on host plants in a laboratory inoculation experiment on 10 *Peronospora sparsa* strains and 20 plant genotypes belonging to *Rubus arcticus*, *R. chamaemorus*, and *R. saxatilis*.

Effect	g–Infection success				h–Speed to sporulation				i–Sporulation abundance				j–Sporulation abundance			
	Num DF	Den DF	*F*	*p*	Num DF	Den DF	*F*	*p*	Num DF	Den DF	*F*	*p*	Num DF	Den DF	*F*	*p*
Strain	**1**	**171**	**2.24**	**0.022**	9	87	1.19	0.3109	9	87	0.78	0.637	–	–	–	–
Species	2	**171**	0	0.9988	2	87	1.54	0.2197	2	87	1.49	0.2307	**2**	**84**	**5.81**	**0.0043**
Genotype within species	**17**	**171**	**1.73**	**0.0403**	**15**	87	**1.94**	**0.0294**	**15**	87	**2.91**	**0.0009**	**15**	**84**	**2.28**	**0.0093**
Speed to sporulation													**1**	**84**	**5.1**	**0.0265**
Speed to sporulation × species													**2**	**84**	**5.21**	**0.0073**

*Note*: The results were analyzed with generalized linear (mixed) models. Statistically significant (*p* < 0.05) results are shown in bold. The letters g–j refer to the models described in Table 1.

### 3.4 Life‐history trait correlation results

**FIGURE 5:**
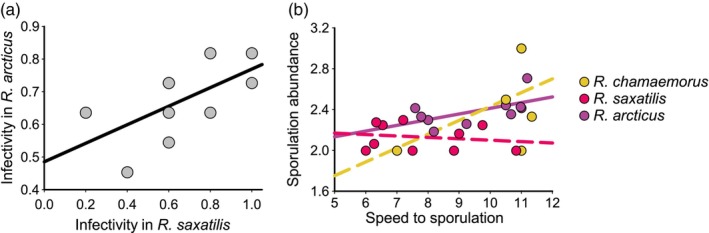
*Peronospora sparsa* life‐history trait correlations in three host plants in an inoculation experiment. (a) Correlation between speed to sporulation and sporulation abundance in *Rubus arcticus*, *R. chamaemorus*, and *R. saxatilis*. (b) Correlation between *P. sparsa* strain infectivity among the host species. Significant correlations are indicated with solid lines.

When potential life‐history trade‐offs limiting pathogen evolution among host species were tested, only positive or non‐significant correlations between life‐history traits were observed. There was a significant positive relationship between infectivity in *R. arcticus* and *R. saxatilis* (*p* = 0.036; *R* ^2^ = 0.43; Figure 5a). Relationships between infectivity in *R. chamaemorus* and both *R. arcticus* (*p* = 0.1268; *R* ^2^ = 0.27) and *R. saxatilis* (*p* = 0.3528; *R* ^2^ = 0.11) were non‐significant. As an alternative approach to understanding pathogen life‐history evolution among host species, I tested for an association between speed to sporulation and sporulation abundance within each host species. I found that sporulation abundance was explained by both host species and host genotype within species as well as speed to sporulation (Table 4). The relationship between sporulation abundance was dependent on host species (Table 4; Figure 5b), such that the positive association between fast and abundant sporulation observed in *R. arcticus* was not observed in *R. saxatilis* or *R. chamaemorus*.

## 4 DISCUSSION

Despite the commonness of generalism in pathogens (Woolhouse et al., 2001) and the risks of emergence of new generalist pathogens of humans due to increased wildlife encounters (Woolhouse et al., 2005), their evolution and epidemiology have gained surprisingly limited attention (Rigaud et al., 2010). Here, I investigated disease prevalence and severity, transmission, and potential life‐history trade‐offs of *P. sparsa* in its three host species *R. arcticus*, *R. chamaemorus*, and *R. saxatilis* across Finland. In an epidemiological survey, I found that disease severity in the three host species was highest in *R. chamaemorus* and lowest in *R. saxatilis*. Disease severity was furthermore higher in larger host populations. Results from a transmission experiment showed that the host species combination in local populations shapes the transmission dynamics of *P. sparsa*. Results from an inoculation experiment confirmed that all three plant species are susceptible to the pathogen and that variation in susceptibility to *P. sparsa* is distributed principally among host genotypes rather than host species. Furthermore, positive life‐history correlations found in *R. arcticus* were altered in *R. saxatilis*, suggesting a possible mechanism of evolutionary constraints that make *R. saxatilis* a suboptimal host for the pathogen (Papaix et al., 2015; Saikkonen et al., 2016).

A better understanding of life‐history traits and the reservoir hosts of pathogens is needed to predict and prevent the emergence of pathogens at the interface of wild and cultivated taxa (Morris et al., 2022). While it is known that the pathogens may freely move across wild and domesticated hosts and even across species borders (Burdon et al., 2006), the data on generalist pathogen epidemics remain scarce (Rigaud et al., 2010). Understanding how pathogen life‐history allocations are altered at host species boundaries will help us to understand the factors that limit the emergence of novel highly virulent strains (Papaix et al., 2015). Most of the research thus far has focused on the spillover of pathogens from cultivated plants to wild plants (Power & Mitchell, 2004). Wild plants serving as reservoir hosts for agricultural pathogens can amplify epidemics and enhance overwintering survival (Power & Mitchell, 2004). Here, in *R. arcticus* and *R. chamaemorus*, I found no difference between wild and cultivated hosts in disease severity. This is in contrast with the expectation that wild host populations are better adapted to their pathogens (Burdon & Thrall, 2009). However, *R. saxatilis* exhibited lower symptoms during the same survey period.

Plant community composition was linked to transmission dynamics in the transmission experiment. Among sites with different host combinations and plant diversities, the trap plants were more frequently infected and had more infected leaves in sites with low plant diversity and large *R. saxatilis* coverage. However, in sites with large *R. saxatilis* coverage, increasing plant diversity decreased disease pressure. This is in line with the expectations of the dilution hypothesis and observations in other plant systems (Haas et al., 2011; Liu et al., 2020; Susi & Laine, 2021). In a study by Haas et al. (2011) on the oomycete pathogen *Phytophthora ramorum*, only two competent host species contributed to epidemics despite the pathogen's wide host range. An alternative explanation for disease variation among plant communities with different compositions is that in a high‐quality environment, hosts may exhibit more severe symptoms (Kniskern & Rausher, 2006). It is possible that sites with a higher *R. saxatilis* coverage may also be more environmentally favorable for *P. sparsa*. However, there were no correlations between the other available site explanatory factors that could explain the observed pattern. The results do, however, suggest that maintaining biodiversity is important in preventing the transmission of generalist pathogens.

There was a strong effect of host genotype on infectivity and sporulation traits in the inoculation experiment and in explaining infection occurrence and severity in the trap plant experiment. This is not surprising given the fact that host characteristics are expected to play a key role in defining infection outcomes (Francl, 2001; Sallinen et al., 2020). The pathogen strains differed both qualitatively, that is, had different infectivity profiles, and quantitatively, that is, varied in the number of host genotypes infected. However, they did not differ in traits related to transmission potential. Symptom occurrence and severity were highest in *R. chamaemorus* in the field sites. However, in the inoculation experiment, two of the four *R. chamaemorus* genotypes were resistant to all *P. sparsa* strains. There are at least two possible explanations for this pattern. First, the higher disease pressure in *R. chamaemorus* populations may lead to the evolution of resistance in this species. Secondly, the pathogen strains used in the inoculation experiment originated from *R. arcticus*. It is possible that strains that naturally infect *R. chamaemorus* differ from those that typically infect *R. arcticus*.

Trade‐offs are expected to limit the evolution and spread of generalist pathogens. Here, I found a positive correlation between pathogen infectivity in *R. saxatilis* and *R. arcticus*. This suggests that trade‐offs do not limit the infectivity of the pathogen between these two closely related species. However, there was no correlation between infectivity in more distantly related *R. chamaemorus* and that in either *R. arcticus* or *R. saxatilis*. The three *Rubus* taxa commonly co‐occur allowing pathogens to circulate in all of them. Thus, an interesting open question for future research is exploration of the genetic basis of resistance in these alternative host species. Strains originating from sites with a high *R. saxatilis* coverage were furthermore less infectious than strains originating from sites without *R. saxatilis* (data not shown). This suggests that the pathogen may incur costs of surviving in more heterogeneous host populations that reduce its ability to infect a range of hosts (Combes, 1997).

Life‐history trade‐offs may operate on pathogen traits important for transmission to hinder the spread of the pathogen in alternative hosts (Papaix et al., 2015; Saikkonen et al., 2016). In this study, when all host plants were considered, there were no significant correlations between the selected two proxies for pathogen fitness: speed to sporulation or abundance. However, when correlations between these two life‐history traits were quantified within each host species, a positive correlation was found in *R. arcticus* but not in *R. saxatilis* or *R. chamaemorus*. All the *P. sparsa* strains used in the study originated from *R. arcticus*. Hence, this result suggests that fast sporulation does not necessarily lead to a high sporulation abundance in alternative hosts. This may be one mechanism for curbing epidemics in populations that contain alternative hosts.

Pathogens may exist unnoticed in wild plants due to their mild symptoms (Prendeville et al., 2012). However, surveying the presence of the symptoms and the microbiota causing these in wild plants is essential for understanding the epidemiology and evolution of pathogen emergence. Linking a pathogen's host range, distribution, and realized symptom severity will allow more robust predictions of the risk of disease emergence. These results shed light on resistance variation among host species. By showing that previously unknown reservoir hosts may have significant impact on epidemiology and evolution of a multi‐host pathogen, this study increases our understanding of the epidemiology of infectious diseases. When bringing novel crops into cultivation, potential risks arising from related plant species that harbor pathogens should be monitored and evaluated systematically. With the rise of novel sequencing methodologies that allow screening of microbial taxa, particular attention should be paid to the screening of the microbiota of wild host plants in order to identify potential disease risks.

## CONFLICT OF INTEREST STATEMENT

The author has declared no competing interests.

## Supporting information


Data S1:

## Data Availability

The data that support the findings of this study are openly available in the Dryad Digital Repository: http://doi.org/10.5061/dryad.vdncjsz32 (Susi, 2024).
